# Supplementary material for: Digital competence of faculty members in health sciences measured via self-reflection: current status and contextual aspects
Source: PeerJ. 2024 Nov 11;12:e18456. doi: 10.7717/peerj.18456 (PMC11562777; doi:10.7717/peerj.18456)
Supplement: Supplemental Information 1 [file peerj-12-18456-s001.pdf]

Digital Competency Assessment Questionnaire for Academicians

This questionnaire has been prepared within the scope of the Erasmus+ project named XXXXXXXXX - (PROJECT NAME AND ID ARE REMOVED FOR BLINDED REVIEW.) (XXXXXXXXXXXXX) in order to determine the self-assessed digital competencies of academicians.

There are two sections;
A. DigCompEdu CheckIn Questionnaire ©*
B. Demographic Questions

All data obtained from this questionnaire will be kept confidential within the scope of the project, the identities or personal information of the participants will be kept confidential and will not be published. Participation in the study is on a voluntary basis.

Thank you for support by participating the questionnaire.

... on behalf of the project team

~~* Indicates that it is compulsory to answer.~~

*Copyright Notice
DigCompEdu is authorised under Creative Commons Attribution 4.0 International licence (CC BY 4.0).

First published, in English, in 2017, as "European Framework for the Digital Competence of Educators: DigCompEdu" by the European Commission's Joint Research Centre, EUR 28775 EN, ISBN 978-92-79-73494-6, doi:10.2760/159770, JRC107466, http://europa.eu/!gt63ch.

This translation is the responsibility of (insert name or organisation.....).

The European Commission is not responsible for this translation and cannot be held liable for any consequence stemming from its use.

Section A: DigCompEdu CheckIn Questionnaire

This self-assessment questions are based on the European Digital Competence Framework for Educators (DigCompEdu). DigCompEdu sets out 22 competences organised in six Areas. The competences are explained at six different levels of proficiency (A1, A2, B1, B2, C1, C2). DigCompEdu addresses educators at all levels of education, from pre-primary to vocational, higher and adult education. The focus of the framework is to support and encourage teachers in using digital tools to enhance and innovate education.

This tool aims to allow you to reflect on your strengths and weaknesses in using digital technologies in education. We invite you to self-assess yourself against 22 items of six areas that are representative for the 22 competences in "DigCompEdu" (https://ec.europa.eu/jrc/en/digcompedu). For each of these items, choose one of five answer options.

1. 1. Your Email Address:

   In order to provide your personal proficiency level

   ____

2. 2. How do you currently assess your digital competence as an academic teaching in higher or further education? *

   Select only one option.

   - A1. Newcomer
   - A2. Explorer
   - B1. Integrator
   - B2. Expert
   - C1 Leader
   - C2. Pioneer

Area #1: Professional Engagement

Academics' digital competence is expressed in their ability to use digital technologies not only to enhance teaching, but also for their professional interactions with colleagues, students, the scientific community and other interested parties, for their individual professional development and for the collective good and continuous innovation in the organisation. This is the focus of Area 1.

Please consider where you stand in view of the following long-term goals.

The answer options are organised by increasing level of engagement with digital technologies. Please choose the option that best reflects your current practice.

3. 1) I systematically use different digital channels to enhance communication with students and fellow academics *

e.g. emails, blogs, the department's website, Apps

Select only one option.

- I rarely use digital communication channels
- I use basic digital communication channels, e.g. e-mail
- I combine different communication channels, e.g. e-mail and class blog or the daprtment's website
- I systematically select, adjust and combine different digital solutions to communicate effectively
- I re ect on, discuss and proactively develop my communication strategies

4. 2. I use digital technologies to work together with colleagues inside and outside my educational organisation *

Select only one option.

- I rarely have the opportunity to collaborate with other academics
- Sometimes I exchange materials with colleagues, e.g. via e-mail
- Among colleagues, we work together in collaborative environments or use shared drives
- I exchange ideas and materials, also with academics outside my organisation, e.g. in an online professional network
- I jointly create materials with other academics in an online network

5. 3. I actively develop my digital teaching skills *

Select only one option.

- I rarely have the time to work on my digital teaching skills
- I improve my skills through reflection and experimentation
- I use a range of resources to develop my digital teaching skills
- I discuss with peers how to use digital technologies to innovate and improve educational practice.
- I help colleagues in developing their digital teaching strategies.

6. 4. I participate in online training opportunities *

e.g. online courses, MOOCs, webinars, virtual conferences...

Select only one option.

- This is a new area that I have not yet considered
- Not yet, but I am definitely interested
- I have participated in online training once or twice
- I have tried out various different online training opportunities
- I frequently participate in all kinds of online training

Area 2: Digital Resources

One of the key competences any academic teaching in higher or further education needs to develop is to identify good educational resources, and to modify, create and share digital resources that fit their learning objectives, student group and teaching style. At the same time they need to be aware of how to responsibly use and manage digital content, respecting copyright rules and protecting personal data. These issues are at the heart of Area 2.

Please consider where you stand in view of the following long-term goals.

The answer options are organised by increasing level of engagement with digital resources. Please choose the option that best reflects your current practice.

7. 1. I use different Internet sites and search strategies to find and select a range of different digital resources *

Select only one option.

- I only rarely use the internet to find resources
- I use search engines and resource platforms to find relevant resources
- I evaluate and select resources on the basis of their suitability for my learner group
- I compare resources using a range of relevant criteria, e.g. reliability, quality, fit, design, interactivity, appeal
- I advise colleagues on suitable resources and search strategies

8. 2. I create my own digital resources and modify existing ones to adapt them to my needs *

Select only one option.

- I do not create my own digital resources
- I do create lecture notes or reading lists with a computer, but then I print them
- I create digital presentations, but not much more
- I create and modify different types of resources
- I set up and adapt complex, interactive resources

9. 3. I effectively protect sensitive content, e.g. exams, students' grades, personal data *

Select only one option.

- I do not need to do that, because the department takes care of this
- I avoid storing personal data electronically
- I protect some personal data
- I password protect files with personal data
- I comprehensively protect personal data, e.g. combining hard-to-guess passwords with encryption and frequent software updates

Area 3: Teaching and Learning

The most fundamental competence of the whole DigCompEdu framework is to design, plan and implement the use of digital technologies in the different stages of the teaching and learning process. However, when doing this, the aim must be to shift the focus from teacher-led to student-centred processes. This is the real power of digital technologies and the focus of Area 3.

Please consider where you stand in view of the following long-term goals.

The answer options are organised by increasing level of engagement with digital technologies in teaching and learning. Please choose the option that best reflects your current practice.

10. 1. I carefully consider how, when and why to use digital technologies in teaching, to ensure that they are used with added value *

Select only one option.

- I do not or only rarely use technology in class
- I make basic use of available equipment, e.g. digital whiteboards or projectors
- I use a variety of digital strategies in my teaching
- I use digital tools to systematically enhance teaching
- I use digital tools to implement innovative pedagogic strategies

11. 2. I monitor my students' activities and interactions in the collaborative online environments we use *

Select only one option.

- I do not use digital environments with my students
- I do not monitor student activity in the online environments we use
- I occasionally check on them and their discussions
- I regularly monitor and analyse my students' online activity
- I regularly intervene with motivating or corrective comments

12. 3. When my students work in groups or teams, they use digital technologies to acquire and document evidence *

Select only one option.

- My students do not work in groups
- It is not possible for me to integrate digital technologies into group work
- I encourage students working in groups to search for information online or to present their results in digital format
- I require students working in teams to use the internet to find information and present their results in a digital format
- My students exchange evidence and jointly create knowledge in a collaborative online space

13. 4. I use digital technologies to allow students to plan, document and monitor their learning themselves *

E.g. quizzes for self-assessment, ePortfolios for documentation and showcasing, online diaries/blogs for re ection...

Select only one option.

- Not possible in my work environment
- My students do reflect on their learning, but not with digital technologies
- Sometimes I use, for example, quizzes for self-assessment
- I use a variety of digital tools to allow learners to plan, document or reflect on their learning
- I systematically integrate different digital tools to allow learners to plan, monitor and reflect on their progress

Area 4: Assessment

Digital technologies can enhance existing assessment strategies and give rise to new and better assessment methods. Additionally, by analysing the wealth of (digital) data available on individual student's (inter-)actions, more targeted feedback and support can be offered. Area 4 addresses this shift in assessment strategies.

Please consider where you stand in view of the following long-term goals.

The answer options are organised by increasing level of engagement with digital assessment. Please choose the option that best reflects your current practice.

14. 1. I use digital assessment formats to monitor student progress *

Select only one option.

- I do not monitor students' progress
- I do monitor students' progress regularly, but not with digital means
- Sometimes I use a digital tool, e.g. a quiz, to check on students' progress
- I use a variety of digital tools to monitor student progress
- I systematically use a variety of digital tools to monitor student progress

15. 2. I analyse all data available to me to timely identify students who need additional support *

"Data" includes: students' engagement, performance, grades, attendance; activities and social interactions in (online) environments;"Students who need additional support" are: students who are at risk of dropping out or underperforming; students who have learning disorders or specific learning needs, students who lack transversal skills, e.g. social, verbal or study skills.

Select only one option.

- These data are not available and/or it is not my responsibility to analyse them
- I only analyse academically relevant data, e.g. performance and grades
- I also consider data on student activity and behaviour to identify students who need additional support
- I regularly screen all available evidence to identify students who need additional support
- I systematically analyse data and intervene in a timely manner

16. 3. I use digital technologies to provide effect ve feedback *

Select only one option.

- Feedback is not necessary in my work environment
- I do provide feedback to students, but not in digital format
- Sometimes I use digital ways of providing feedback, e.g. automatic scores in online quizzes, comments or "likes" in online environments
- I use a variety of digital ways of providing feedback
- I systematically use digital approaches to provide feedback

Area 5: Empowering Learners

One of the key strengths of digital technologies in education is their potential for boosting the active involvement of students in the learning process and their ownership of it. Digital technologies can furthermore be used to offer learning activities adapted to each individual student's level of competence, their interests and learning needs. At the same time, however, care must be taken not to exacerbate existing inequalities and to ensure accessibility for all students. Area 5 tackles these issues.

Please consider where you stand in view of the following long-term goals.

The answer options are organised by increasing focus on students' individual learning needs. Please choose the option that best reflects your current practice.

17. 1. When I create digital assignments for students I consider and address potential digital problems *

E.g. equal access to digital devices and resources; interoperability and conversion problems; lack of digital skills

Select only one option.

- I do not create digital assignments
- My students do not have problems with using digital technology
- I adapt the task so as to minimize difficulties
- I discuss possible obstacles with students and outline solutions
- I allow for variety, e.g. I adapt the task, discuss solutions and provide alternative ways for completing the task

18. 2. I use digital technologies to offer students personalised learning opportunities*

e.g. I give different students different digital tasks to address individual learning needs, preferences and interests

Select only one option.

- In my work environment, all students are required to do the same activities, irrespective of their level
- I do provide students with recommendations for additional resources
- I provide optional digital activities for those who are advanced or lagging behind
- Whenever possible, I use digital technologies to offer differentiated learning opportunities
- I systematically adapt my teaching to link to students' individual learning needs, preferences and interests

19. 3. I use digital technologies for students to act vely part c pate n classes *

Select only one option.

- In my work environment it is not possible to actively involve students in class
- I do involve students actively, but not with digital technologies
- When instructing, I use motivating stimuli, e.g. videos, animations, cartoons
- My students engage with digital media in my classes, e.g. electronic worksheets, games, quizzes
- My students systematically use digital technologies to investigate, discuss and create knowledge

Area 6: Facilitating Learners' Digital Competence

The ability to facilitate students' digital competence is an integral part of academics' digital competence and at the heart of Area 6.

Please consider where you stand in view of the following long-term goals.

The answer options are organised by increasing level of engagement in fostering students' digital competence. Please choose the option that best reflects your current practice.

20. 1. I teach students how to assess the reliability of information and to identify mis information and bias *

Select only one option.

- This is not possible in my subject or work environment
- I occasionally remind them that not all online information is reliable
- I teach them how to discern reliable and unreliable sources
- I discuss with students how to verify the accuracy of information
- We comprehensively discuss how information is generated and can be distorted

21. 2. I set up assignments which require students to use digital means to communicate and collaborate with each other or with an outs de aud ence *

Select only one option.

- This is not possible in my subject or work environment
- Only on rare occasions are my students required to communicate or collaborate online
- My students use digital communication and cooperation mainly among each other
- My students use digital ways to communicate and to cooperate with each other and with an external audience
- I systematically set assignments that allow students to slowly expand their skills

22. 3. I set up assignments which require students to create digital content *

e.g. videos, audios, photos, digital presentations, blogs, wikis...

Select only one option.

- This is not possible in my subject or work environment
- This is difficult to implement with my students
- Sometimes, for diversion and motivation
- My students create digital content as integral part of their study
- This is an integral part of their learning and I systematically increase the level of difficulty to further develop their skills

23. 4. I teach students how to behave safely and responsibly online *

Select only one option.

- This is not possible in my subject or work environment
- I inform them that they have to be careful with relaying personal information online
- I explain the basic rules for safely and responsibly acting in online environments
- We discuss and agree on rules of conduct
- I systematically develop my students' use of social rules in the different digital environments we use

24. 5. I encourage students to use digital technologies creatively to solve concrete problems *

e.g. to overcome obstacles or challenges emerging in the learning process

Select only one option.

- This is not possible with my students, in my work environment
- I rarely have the opportunity to foster students' digital problem solving
- Occasionally, whenever an opportunity arises
- We often experiment with technological solutions to problems
- I systematically integrate opportunities for creative digital problem solving

Section B: Demographic Questions:

…some questions about you

To improve this questionnaire, we would like to ask you some questions about yourself. These questions are only used by scientists to better understand for which groups of users this questionnaire makes sense - and for which perhaps not.

25. 1. Academic Title:

* *Select only one option.*

- Prof. Dr.
- Assoc. Prof. Dr.
- Assist. Prof. Dr.
- Research Assistant
- Lecturer

26. 2. Your gender:

* *Select only one option.*

- Female
- Male
- Prefer not to say

27. 3. Your age: *

____

28. 4. Teaching experience in higher education in years: *

____

29. 5. In which of the following subject areas do you teach? *

Multiple answers are possible

- [ ] Nursing
- [ ] Nutrition and Dietetics
- [ ] Physiotherapy and Rehabilitation
- [ ] Audiology
- [ ] Healthcare Management
- [ ] Social Work
- [ ] Exercise and Sport Sciences
- [ ] Occupational Therapy
- [ ] Other: ______________________

30. 6. What is the main profile of the students you teach? *

Multiple answers are possible

- [ ] Undergraduate students
- [ ] Graduate master students
- [ ] Graduate doctorate students
- [ ] Adult students full-time
- [ ] Adult students part-time
- [ ] Other: ______________________

DT in teaching

31. 7. How long have you been using DT* in teaching ? (Indicate in years / zero for never) *

*Digital Technology (DT) means all information and communication technologies such as mobile phone, computer, Internet, software etc. that can be used to create, view, distribute, modify, store, retrieve, transmit and receive information electronically in a digital form.

____

32. 8. Which digital tools have you or your students already used for teaching and learning?

Multiple answers are possible

- [ ] Presentations
- [ ] Digital posters, mindmaps, planning tools
- [ ] Watching videos / listening to audios
- [ ] Digital quizzes or polls (ie Kahoot, Mentimeter, etc)
- [ ] Blogs or wikis
- [ ] Creating videos / audios
- [ ] Online/Virtual learning environments (Moodle, etc)
- [ ] Online communication tools (Zoom, Teams, Skype, Google Meet, etc)
- [ ] I have not yet used any digital tools in class
- [ ] Other: ______________________

33. 9. Do you have the following digital technologies for teaching / learning provi ded by your university? *

Multiple answers are possible

- [ ] Learning Management System or Virtual Learning Environment (like Moodle/Blackboard- etc.) (Should we get info about LMS names from each partner for this question)
- [ ] Student Enrolment and Grading System (course lists, grading, attendance, etc.)
- [ ] Asynchronous Communication Tools (Email, SMS, Messaging)
- [ ] Synchronous Communication Tools (Zoom, Teams, Skype etc.)
- [ ] Online or electronic measurement and evaluation systems
- [ ] Other: ______________________

34. 10. Do you have advanced or field-specific DT for teaching / learning? *

Multiple answers are possible

- [ ] Augmented/Virtual Reality Tools or environments
- [ ] Simulations
- [ ] Software or applications for specific tasks (like for advanced calculation, data analysis, etc.)
- [ ] Special institutional membership for certain online collections / databases
- [ ] Diğer: ______________________

35. 11. Do you have any experience in teaching online? *

Multiple answers are possible

- [ ] Yes, I've given a fully online course only during the Covid pandemic
- [ ] Yes, I've given a fully online course before the Covid pandemic
- [ ] Yes, I've given a partially online course (in blended or hybrid form) only during the Covid Pandemic
- [ ] Yes, I've given a partially online course (in blended or hybrid form) before the Covid Pandemic
- [ ] No, I have never given an online course
- [ ] Diğer: ______________________

36. 12. Do you have any exper ence n part c pat ng/enroll ng an onl ne course or cert f cate programme? *

Multiple answers are possible

- [ ] Yes, I've participated/enrolled a fully online course/certificate programme
- [ ] Yes, I've participated/enrolled a partially online (in blended or hybrid form) course/certificate programme
- [ ] No, I don't have such learning experience

Private usage of DT

37. 13. How would you describe yourself and your private use of digital technologies? *

Select only one option in each row.

	Strongly disagree	Disagree	Neither agree or disagree	Agree	Strongly agree
**I find it easy to work with computers and other technical equipment**	⬭	⬭	⬭	⬭	⬭
**I use the Internet extensively and competently**	⬭	⬭	⬭	⬭	⬭
**I am open and curious about new apps, programs, resources**	⬭	⬭	⬭	⬭	⬭
**I am a member of various social networks**	⬭	⬭	⬭	⬭	⬭

38. 14. How well does your work environment meet the following criteria? *

Select only one option in each row.

	Strongly disagree	Disagree	Neither agree or disagree	Agree	Strongly agree
**The department invests in updating and improving the technical infrastructure.**					
**The department provides the necessary technical support.**					
**Students have access to digital devices**					
**The internet connection of the department is reliable and fast.**					
**The department supports the development of my digital competence, e.g. through continuous professional development activities.**					

39. 15. How would you now, after responding to the questionnaire, assess your digital competence as an academic teaching in higher or further education? *

Assign a level of competence from A1 to C2, where A1 is the lowest and C2 the highest level.

Select only one option.

- A1: Newcomer
- A2: Explorer
- B1: Integrator
- B2: Expert
- C1: Leader
- C2: Pioneer

Google Formlar
